# Bulletin of Medical Science

**Published:** 1847-04

**Authors:** 


### ARTICLE IX.

### BULLETIN OF MEDICAL SCIENCE.

This valuable exchange has been discontinued. Few men in this country are as able with the pen as Dr. Bell, and his retirement from the editorial chair is a loss to the profession.

